# A Genetic Screen for Fission Yeast Gene Deletion Mutants Exhibiting Hypersensitivity to Latrunculin A

**DOI:** 10.1534/g3.116.032664

**Published:** 2016-07-27

**Authors:** Farzad Asadi, Dorothy Michalski, Jim Karagiannis

**Affiliations:** Department of Biology, University of Western Ontario, London, Ontario, Canada, N6A 5B7

**Keywords:** *Schizosaccharomyces pombe*, latrunculin A, cytokinesis, actin, cell division, Mutant Screen Report

## Abstract

Fission yeast cells treated with low doses of the actin depolymerizing drug, latrunculin A (LatA), delay entry into mitosis via a mechanism that is dependent on both the Clp1p and Rad24p proteins. During this delay, cells remain in a cytokinesis-competent state that is characterized by continuous repair and/or reestablishment of the actomyosin ring. In this manner, cells ensure the faithful completion of the preceding cytokinesis in response to perturbation of the cell division machinery. To uncover other genes with a role in this response, or simply genes with roles in adapting to LatA-induced stress, we carried out a genome-wide screen and identified a group of 38 gene deletion mutants that are hyper-sensitive to the drug. As expected, we found genes affecting cytokinesis and/or the actin cytoskeleton within this set (*ain1*, *acp2*, *imp2*). We also identified genes with roles in histone modification (*tra1*, *ngg1*), intracellular transport (*apl5*, *aps3*), and glucose-mediated signaling (*git3*, *git5*, *git11*, *pka1*, *cgs2*). Importantly, while the identified gene deletion mutants are prone to cytokinesis failure in the presence of LatA, they are nevertheless fully capable of cell division in the absence of the drug. These results indicate that fission yeast cells make use of a diverse set of regulatory modules to counter abnormal cytoskeletal perturbations, and furthermore, that these modules act redundantly to ensure cell survival and proliferation.

Latrunculin A (LatA) is a naturally occurring macrolide toxin produced by the red sea sponge, *Negombata magnifica* (formerly known as *Latrunculia magnifica*) (Spector *et al.* 1983). The drug, which consists of a 16-member lactone ring attached to a 2-thiazolidinone moiety, acts within living cells by sequestering G-actin, thereby preventing F-actin assembly (Spector *et al.* 1983; Coué *et al.* 1987; Yarmola *et al.* 2000). *In vitro* studies have shown that LatA binds monomeric actin with a 1:1 stoichiometry and has an equilibrium dissociation constant (K_d_) of only ∼0.2 μM (Coué *et al.* 1987; Yarmola *et al.* 2000). When used at high concentrations that are well above its K_d_ (*i.e.*, 10–50 μM), it is effective in completely disrupting the actin cytoskeleton over a ime-frame of minutes (Ayscough 1998). This property, together with the fact that it is fully cell permeable, has made LatA a popular tool in studies aimed at elucidating the biological roles of the actin cytoskeleton.

While typically used at high concentrations, LatA has also been used at much lower levels (*i.e.*, near the equilibrium constant of 0.2 μM) to mildly perturb the actin cytoskeleton (Coué *et al.* 1987). In fission yeast, for example, such low dose treatment disrupts the cell division machinery, leading to the activation of a cytokinesis checkpoint system (Mishra *et al.* 2004). This system promotes the establishment of a cytokinesis-competent state characterized by delayed progression into mitosis and the continuous repair and/or reestablishment of the cytokinetic actomyosin ring (reviewed in Karagiannis 2012). Key regulators of this response are the Cdc14 family phosphatase, Clp1p, and the 14-3-3 protein, Rad24p (Mishra *et al.* 2005; Chen *et al.* 2008).

While dispensable under normal growth conditions, both Clp1p and Rad24p become indispensable when the cell division machinery is perturbed. Under such stresses, Clp1p is actively retained in the cytoplasm until cytokinesis is complete. During this period, Clp1p functions to delay entry into the subsequent mitosis and promote constriction of the actomyosin ring (through positive regulation of the septation initiation network). The cytoplasmic retention of Clp1p is mediated through Rad24p, which recognizes and binds a phosphorylated form of the protein. This Rad24p-dependent retention of Clp1p to the cytoplasm is critical for cell division since, unlike wild-type cells, *clp1*Δ and *rad24*Δ mutants are inviable upon LatA treatment and display a terminal phenotype characterized by multinucleated cells that have repeatedly failed in cytokinesis (Mishra *et al.* 2005; Chen *et al.* 2008).

While a variety of hyper-sensitive mutants have been isolated and characterized (Karagiannis *et al.* 2005; Karagiannis and Balasubramanian 2007; Saberianfar *et al.* 2011; Rentas *et al.* 2012; Grewal *et al.* 2012), a global assessment of the regulatory modules needed to respond to LatA-mediated perturbation is lacking. In this report, we make use of the Bioneer deletion mutant library (Kim *et al.* 2010) to identify genes that play a role in defending against LatA-induced stress on a genome-wide scale. The results of our screen indicate that fission yeast cells make use of a diverse set of regulatory modules, with wide-ranging biological functions, to mitigate the detrimental effects of abnormal cytoskeletal perturbations.

## Materials and Methods

### Primary screen

The primary screen was conducted in triplicate using version 4 of the Bioneer genome-wide deletion mutant library (http://us.bioneer.com/products/spombe/spombeoverview.aspx). The library consists of 3400 haploid gene deletion mutants, comprising ∼95.3% of nonessential *Schizosaccharomyces pombe* genes. Each strain carries a defined gene deletion constructed with the *kanMX4* cassette (Kim *et al.* 2010). To assay LatA sensitivity, the gene deletion mutants were grown in 96-well microtiter plates in liquid YES media (Forsburg and Rhind 2006) at 30°. Five microliter aliquots were then spotted onto YES-agar media containing 0.4 μM LatA (Focus Biomolecules, cat. no. 10-2254) or YES-agar media containing an equivalent volume of DMSO (solvent control). The growth of the strains on LatA plates (relative to DMSO controls) was assayed visually after 3 d at 30°. Hits were categorized into three groups: a “high-confidence” group comprised of gene deletion mutants that were scored as sensitive in each of the three trials, a “medium-confidence” group comprised of gene deletion mutants that were scored as sensitive in two of the three trials, and a “low-confidence” group comprised of gene deletion mutants that were scored as sensitive in only one of the three trials.

### Spot assays

The respective gene deletion mutants of the high-confidence group were grown overnight at 30° in liquid YES media to an OD of 0.5. Five microliters of undiluted culture, as well as four 10-fold serial dilutions (made in liquid YES), were then spotted onto YES-agar plates containing DMSO (solvent control), or 0.1, 0.2, or 0.3 μM LatA. Growth was assayed visually after the plates had been incubated for 4 d at 30°.

### Disk diffusion assays

The respective gene deletion mutants of the high-confidence group were grown overnight at 30° in liquid YES media to an OD of 0.5. Cells at a concentration of 10^5^ cells/ml were then mixed with molten YES-agar (∼40°) and poured into Petri plates. Five microliters of DMSO (solvent control), or 2.2, 4.4, or 6.6 μM LatA, were then spotted onto sterile filter paper disks (Whatmann No. 1), which were subsequently placed onto the surface of the respective YES-agar plates. Photographs were taken using a FluorChem SP imager after the plates had been incubated for 4 d at 30°. The area of the zone of inhibition for each disk was measured using ImageJ software (National Institutes of Health, Bethesda, MD; https://imagej.nih.gov/ij/). The area of the zone of inhibition for each disk (measured in pixels) was then plotted against the LatA concentration. The slope of the linear regression line was calculated using Microsoft Excel.

### Minimum inhibitory concentration assays

The respective gene deletion mutants of the high-confidence group were grown overnight at 30° in liquid YES media to an OD of 0.5. Approximately 10^5^ cells of each mutant were then seeded into the wells of a 96-well microtiter plate containing 100 μl of YES medium with 0, 0.025, 0.05, 0.075, 0.1, or 0.2 μM LatA. The plates were incubated for 4 d at 30°. The minimum inhibitory concentration (MIC) was determined by visually inspecting the plates to ascertain the lowest concentration of LatA that prevented visible growth.

### Fluorescence microscopy

The respective gene deletion mutants of the high-confidence group were grown overnight at 30° in liquid YES media to an OD of 0.3. The cultures were then treated with DMSO or 0.3 μM LatA for 5 hr. Cells were fixed with two volumes of ice-cold ethanol and then spun at 5000 rpm for 2 min and resuspended in 1 ml of PBS (pH 7.4) containing 1% Triton X-100. Cells were washed three times in PBS before being resuspended in 100 µl of PBS containing 15% glycerol. To observe nuclei and cell wall/septa material, cells were mixed with 0.02 mg/ml 4′6-diamidino-2-phenylindole (DAPI) and 1 mg/ml aniline blue. Fluorescence images (DAPI filter set) were obtained with a Zeiss Axioskop 2 microscope attached to a Scion CFW Monochrome CCD Firewire Camera (Scion Corporation, Frederick, MD). The microscope system was driven by ImageJ 1.41 software.

### Gene Ontology enrichment analysis

The Biological Networks Gene Ontology tool (BiNGO) is an open-source software package that can be used to uncover enrichments in Gene Ontology (GO) terms (http://www.psb.ugent.be/cbd/papers/BiNGO/Home.html). The tool is freely available as a Cytoscape plugin and can be downloaded using the Cytoscape app manager (http://www.cytoscape.org/). To identify GO term enrichments (GO Biological Process, GO Cellular Component, and GO Molecular Function), the 288 hits of the primary screen were inputted against a background of the 3400 *S. pombe* genes of the Bioneer gene deletion set and analyzed using a hyper-geometric test. A multiple testing correction (Benjamini-Hochberg false discovery rate, p-value cutoff of 0.005) was applied to all analyses. Networks were visualized with Cytoscape using a hierarchical layout. A detailed description of the statistical methods used by BiNGO can be found in Maere *et al.* (2005).

### Data availability

The authors state that all data necessary for confirming the conclusions presented in the article are represented fully within the article.

## Results and Discussion

### Primary screen

When challenged with low doses of LatA (0.2–0.5 μM), fission yeast mutants defective in the cytokinesis checkpoint system exhibit a terminal phenotype characterized by inviable multinucleate cells that have failed in cytokinesis. In contrast, wild-type cells remain viable under these same conditions and are able to complete cytokinesis, albeit over a longer timeframe than normal (Mishra *et al.* 2004; Mishra *et al.* 2005; Karagiannis *et al.* 2005; Karagiannis and Balasubramanian 2007; Saberianfar *et al.* 2011; Rentas *et al.* 2012). We reasoned that this characteristic LatA-sensitive phenotype could be used as the basis of a screen aimed at identifying novel genes with roles in promoting successful cell division upon perturbation of the cytokinetic machinery. We also reasoned that the screen would prove useful in identifying genes with roles in adapting to LatA-induced cytoskeletal stress in general. We obtained version 4 of the Bioneer gene deletion library (Kim *et al.* 2010) and carried out a screen for deletion mutants exhibiting hyper-sensitivity to LatA. A schematic providing a broad overview of the screen described in this short report is shown in Supplemental Material, Figure S1.

**Figure 1:**
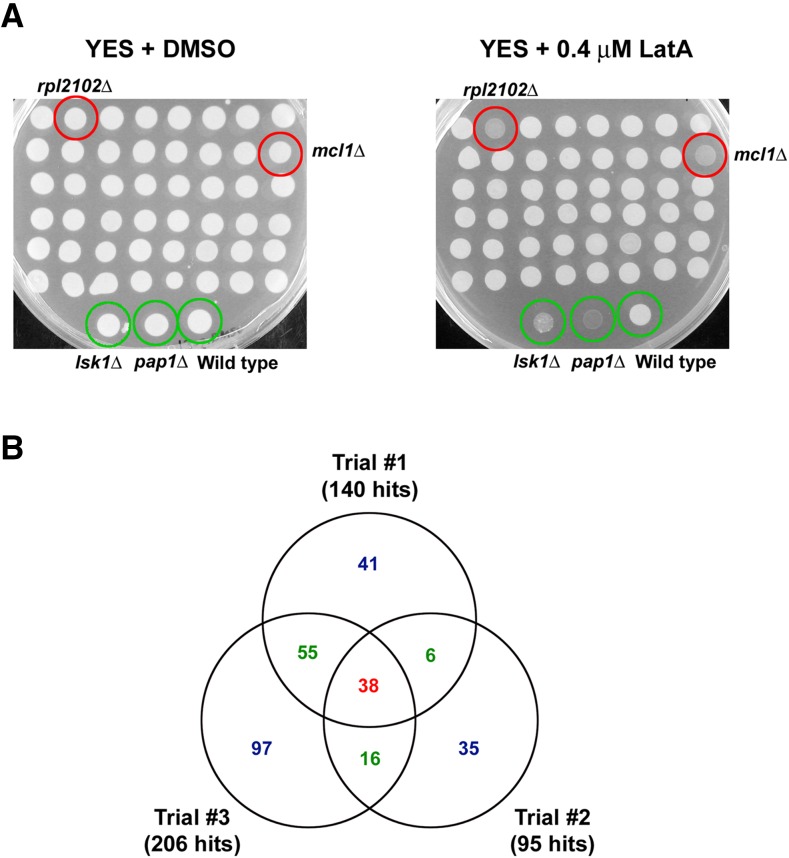
Primary screen. (A) All 3400 gene deletion strains from the Bioneer collection were spotted to YES-agar plates containing DMSO (solvent control) or 0.4 μM LatA and then incubated for 3 d at 30°. Two representative plates from the screen are shown. A wild-type strain, as well as two hyper-sensitive mutants, *lsk1*Δ and *pap1*Δ, were used as controls (green circles). Two “hits,” *rpl2102* and *mcl1*Δ, are also highlighted (red circles). (B) Venn diagram analysis of the three trials of the primary screen. Thirty-eight hits were common to all three trials and comprise the high-confidence group (red). Seventy-seven hits were common to two of the trials and comprise the medium-confidence group (green). One hundred and seventy-three hits were common to one of the three trials and comprise the low-confidence group (blue).

We screened the entire library of 3400 strains by plating the gene deletion mutants on YES media containing 0.4 μM LatA and incubating them for 3 d at 30° (see *Materials and Methods*). Growth was compared to the same mutants plated on YES media containing DMSO (solvent control). To minimize false negatives, the primary screen was performed in triplicate. Furthermore, each trial was carried out blind (*i.e.*, those carrying out the screen were unaware of the genotypes of the strains they were scoring). Three strains were used as controls: *lsk*Δ, a known LatA-sensitive mutant showing low to moderate sensitivity (Karagiannis *et al.* 2005); *pap1*Δ, a mutant known to be hyper-sensitive to a wide variety of toxins (and which we observed to be highly sensitive to LatA; J. Karagiannis and B. Chakraborty, unpublished data); and lastly, a wild-type strain. Representative plates from one trial of the screen are shown in Figure 1A.

After completing the primary screen, we categorized the hits into three groups: a high-confidence group of 38 strains, a medium-confidence group of 77 strains, and a low-confidence group of 173 strains (Figure 1B and File S1). The high-confidence group was comprised of gene deletion mutants that were scored as hits in each of the three trials. The medium-confidence group was comprised of gene deletion mutants that were scored as hits in two of the three trials. Finally, the low-confidence group was comprised of gene deletion mutants that were scored as hits in only one of the three trials. Of eight previously characterized LatA-sensitive mutants present in version 4 of the library, two were present in the low-confidence group (*lsk1*Δ, *hos2*Δ), and six were present in the medium-confidence group (*clp1*Δ, *rad24*Δ, *lsg1*Δ, *sif2*Δ, *snt1*Δ, *set3*Δ).

While the low-confidence group is likely high in false positives, we include the data here for the sake of completeness. Moreover, the fact that both *lsk1*Δ and *hos2*Δ were categorized as low-confidence hits suggests that the group contains at least some true positives. In contrast, the high- and medium-confidence lists, while lower in false positives, must by the same token suffer from higher false negative rates. Thus, by including our complete primary data in its entirety (high-, medium-, and low-confidence lists), we strike the best balance with respect to mitigating the effects of false negative and false positive categorizations and provide the broadest resource possible.

### Secondary screen

**Figure 2:**
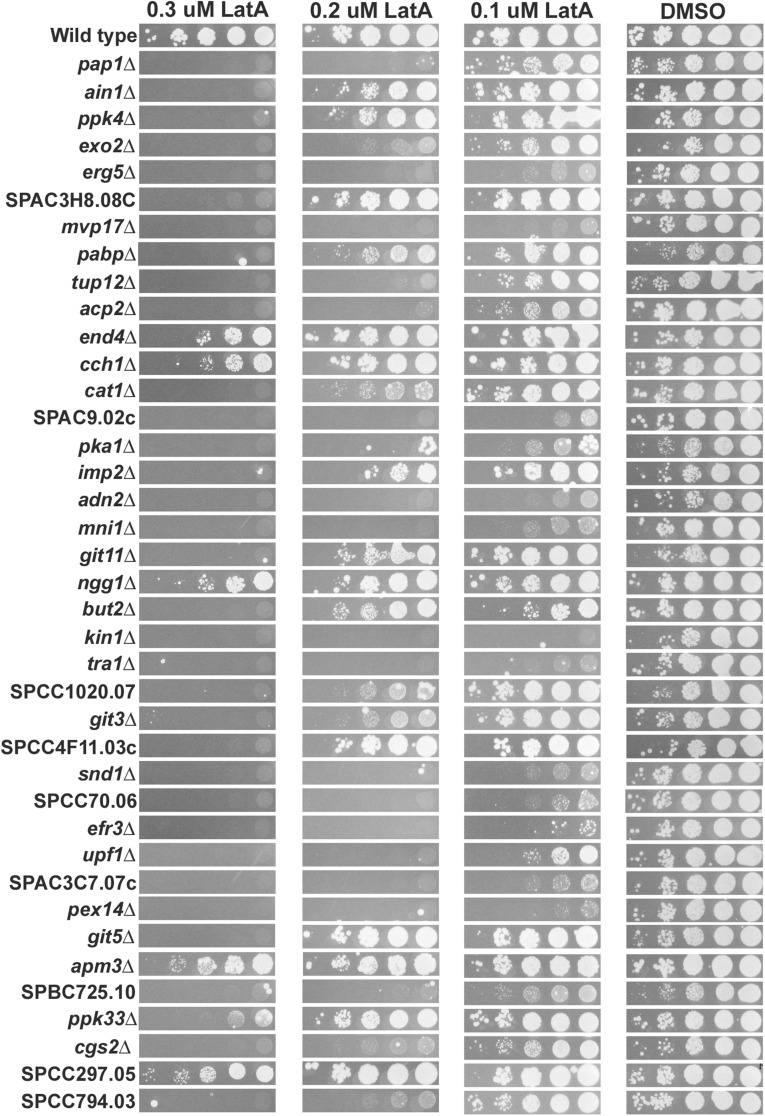
Spot assays. Ten-fold serial dilution of cultures of the indicated genotypes were spotted onto YES-agar plates containing DMSO (solvent control) or 0.1, 0.2, or 0.3 μM LatA. Photographs were taken after 4 d incubation at 30°.

Having completed the primary screen, we next decided to focus on and validate the deletion mutants of the high-confidence group. We began by spotting 10-fold serial dilutions of logarithmically growing cultures of each of the high-confidence hits onto YES medium containing DMSO or 0.1, 0.2, or 0.3 μM LatA. These experiments verified that the mutants were indeed hyper-sensitive to LatA (Figure 2). The sensitivity of the mutants varied from mild (capable of moderate growth on 0.3 μM LatA relative to wild type) to severe (incapable of growth even at 0.1 μM LatA).

**Figure 3:**
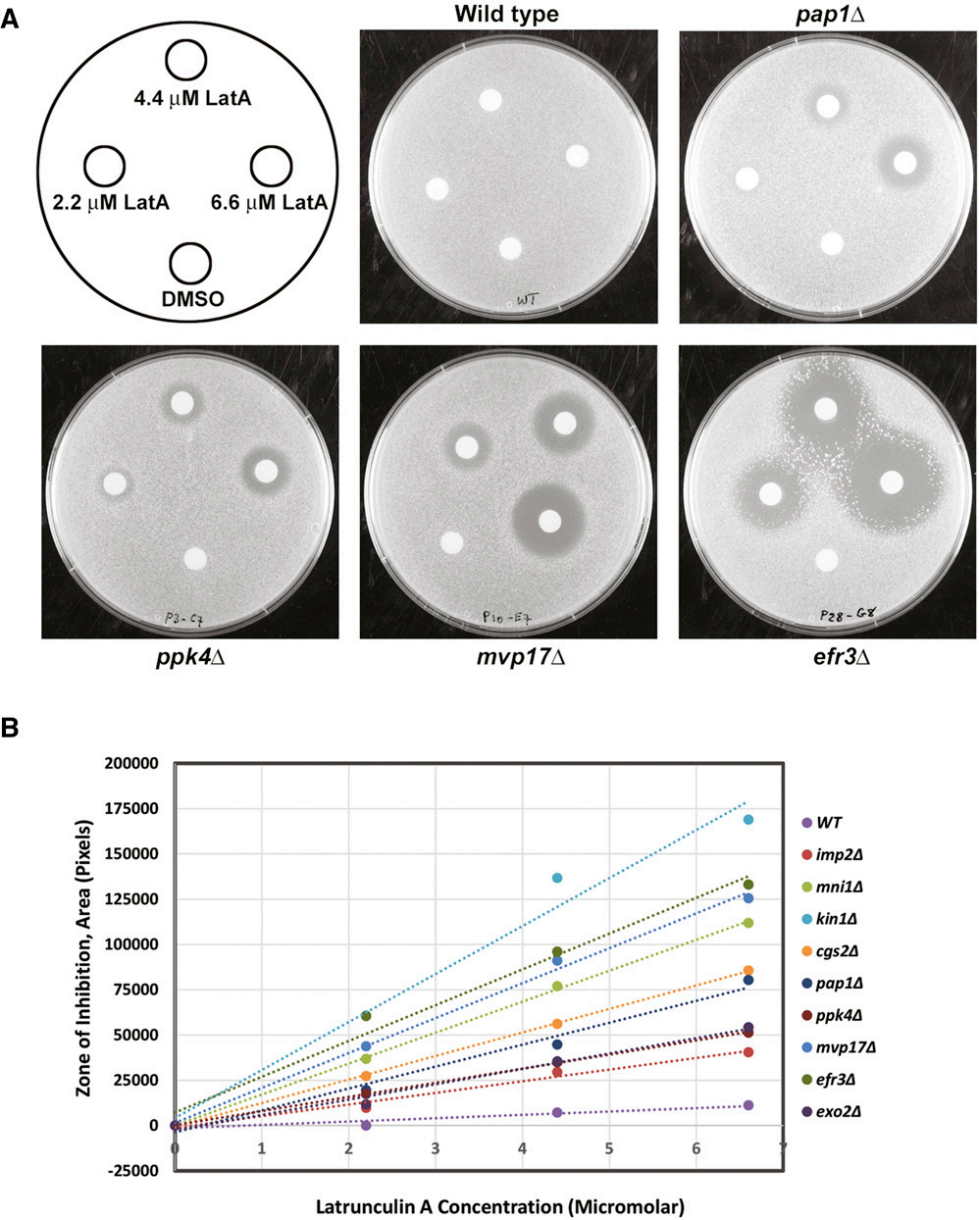
Disk diffusion assays. (A) Filter paper disks soaked with 5 µl of DMSO (solvent control) or 2.2, 4.4, or 6.6 µM LatA, were placed on YES-agar plates impregnated with the indicated gene deletion mutants. Photographs were taken after 4 d incubation at 30°. The zones of growth inhibition surrounding each disk were measured and analyzed as described in *Materials and Methods*. (B) A plot of the area of the zone of inhibition *vs.* LatA concentration for a representative group of 10 gene deletion strains exhibiting varying sensitivity to LatA. The linear regression line through the points is plotted. The slope of this line was used as a quantitative measure to rank the LatA sensitivity of each strain.

Next, to provide a more quantitative assessment of LatA sensitivity, we performed a series of disk diffusion assays (see *Materials and Methods*). In these assays, filter paper disks soaked in DMSO (solvent control) or 2.2, 4.4, or 6.6 μM LatA were placed on YES-agar plates impregnated with the respective gene deletion mutants. The plates were then incubated at 30° for 4 d. Representative plates (*pap1*Δ and wild-type controls, as well as three gene deletion mutants displaying varying levels of sensitivity) are shown in Figure 3A.

For each strain, the area of the zone of inhibition around each disk was plotted against LatA concentration. Linear regression was then used to create a line of best fit through the data points. The slopes of the lines were then calculated. Using this quantitative measure, we were able to both validate the high-confidence hits and accurately rank the strains according to their sensitivity to LatA (Figure 3B and Table 1).

**Table 1:** Ranking of the LatA sensitivity of each gene deletion mutant based on linear regression analysis of data plotting the area of the zone of inhibition (pixels) *vs.* LatA concentration (µM)

Gene Deletion	Rank (Most Sensitive to Least Sensitive)	Slope of the Linear Regression Line (pixels/µM)
*kin1*Δ	1	26,505
*cch1*Δ	2	22,989
*efr3*Δ	3	19,765
*adn2*Δ	4	19,447
*mvp17*Δ	5	19,283
*pex14*Δ	6	17,437
*pka1*Δ	7	17,303
*mni1*Δ	8	17,085
*acp2*Δ	9	16,543
SPCC794.03	10	16,403
SPCC70.06	11	16,360
SPAC9.02c	12	15,836
*erg5*Δ	13	15,748
*tra1*Δ	14	15,388
*snd1*Δ	15	15,216
SPAC3C7.07c	16	14,287
*upf1* Δ	17	13,785
SPCC297.05	18	12,995
*pap1*Δ	19	12,108
*pabp*Δ	20	10,286
*tup12*Δ	21	9763
SPCC4F11.03c	22	9749
*ngg1*Δ	23	9289
*git5*Δ	24	8890
*ain1*Δ	25	8717
*exo2*Δ	26	8480
*cat1*Δ	27	8312
*git3*Δ	28	8062
*ppk4*Δ	29	7802
SPBC725.10	30	7485
SPCC1020.07	31	7064
*git11*Δ	32	6670
*ppk33*Δ	33	6669
*imp2*Δ	34	6424
*apm3*Δ	35	5886
SPAC3H8.08c	36	5555
*end4*Δ	37	4862
*but2*Δ	38	4711
*cgs2*Δ	39	4080
Wild type	40	1852

Lastly, we gauged the mutant’s sensitivity to LatA by determining the MIC for each of the high-confidence hits. This was accomplished using the method of Tebbets *et al.* (2012). Briefly, 10^5^ cells of an overnight culture of each mutant were seeded into the wells of a microtiter plate. Each well contained 100 μl of YES media containing LatA at concentrations ranging from 0.025 to 0.2 μM. The plates were then incubated at 30° for 4 d. The MIC was defined as the lowest concentration of LatA that prevented visible growth. The MICs varied from >0.2 μM for wild-type cells to 0.025 μM for the most sensitive mutants (Table 2). Thus, LatA tolerance is genetically controlled over at least an eightfold range in *S. pombe*.

**Table 2:** Minimum inhibitory concentration of LatA for the indicated gene deletion mutants

Gene Deletion Mutant	Minimum Inhibitory Concentration (µM)
*ain1*Δ	0.1–0.2
*ppk4*Δ	0.2
*exo2*Δ	0.075
*erg5*Δ	0.025–0.075
SPAC3H8.08c	0.2
*mvp17*Δ	0.05
*pabp*Δ	0.1–0.2
*tup12*Δ	0.025
*acp2*Δ	0.1–0.2
*end4*Δ	0.2
*cch1*Δ	>0.2
*cat1*Δ	0.1–0.2
SPAC9.02c	0.075–0.1
*pka1*Δ	0.2
*imp2*Δ	0.025
*adn2*Δ	0.025
*mni1*Δ	0.05
*git11*Δ	0.2
*ngg1*Δ	0.2
*but2*Δ	0.2
*kin1*Δ	0.025
*tra1*Δ	0.05
SPCC1020.07	0.2
*git3*Δ	0.1–0.2
SPCC4F11.03c	0.1
*snd1*Δ	0.075–0.1
SPCC70.06	0.075
*efr3*Δ	0.05–0.075
*upf1*Δ	0.075–0.1
SPAC3C7.07c	0.075–0.1
*pex14*Δ	0.05–0.075
*git5*Δ	0.2
*apm3*Δ	>0.2
SPBC725.10	0.1–0.2
*ppk33*Δ	0.2
*cgs2*Δ	0.075
SPCC297.05	>0.2
SPCC794.03	0.05
*pap1*Δ	0.05–0.075
Wild type	>0.2

### Screening for cytokinesis defects

Having validated the high-confidence hits, we were next interested in better characterizing the nature of each mutant’s sensitivity to LatA. To screen for any obvious cytokinetic phenotypes, we treated the respective mutants with 0.3 μM LatA for 5 hr and then visualized the cell nuclei, as well as cell wall/septal material, by fixing and staining with a mixture of DAPI and aniline blue. The cells were then classified into one of four categories: 1) uninucleate cells, 2) binucleate cells with complete septa, 3) binucleate cells with fragmented/incomplete septa, and 4) tetranucleate cells with fragmented/incomplete septa. We then calculated the ratio of binucleate or tetranucleate cells with fragmented/incomplete septa, to uninucleate cells or binucleate cells with complete septa.

**Figure 4:**
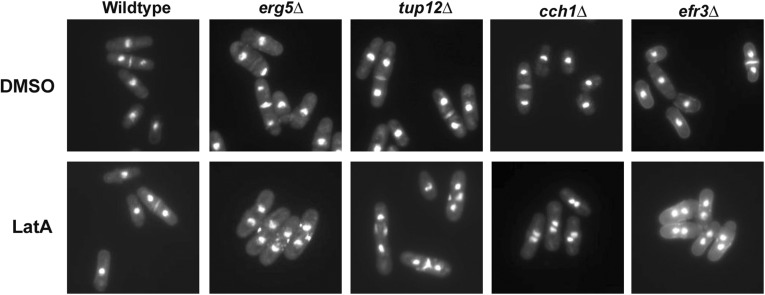
The gene deletion mutants of the high-confidence list are prone to cytokinesis failure in the presence of LatA. Cells of the indicated genotype were grown to midlog phase and then treated with DMSO (solvent control) or 0.3 μM LatA for 5 hr. Cells were fixed and stained with DAPI/aniline blue to visualize nuclei and cell/wall septal material, respectively. Four strains from the high-confidence list are shown as representative examples.

While the wild-type control strain accumulated a majority of cells that were uninucleate, or binucleate with complete septa, the 38 mutants of the high-confidence group accumulated much higher proportions of cells with fragmented/incomplete septa (indicating failed constriction of the actomyosin ring) (Figure 4 and Table 3). In fact, 15 of the 38 mutants (39%) exhibited a majority of cells with fragmented septa. Importantly, although compromised in their ability to complete cytokinesis in the presence of LatA, the high-confidence mutants were nevertheless fully capable of successful cell division in the presence of DMSO. The mutants of the high-confidence group are thus defective in some aspect of their response to LatA-induced cytoskeletal perturbation and are thereby more prone to cell division failure. The mechanism(s) underlying the observed cytokinetic defects of the high-confidence mutants are unknown and will be the subject of future studies.

**Table 3:** Mean percentage of cells (± SD) displaying the indicated phenotype after 5 hr treatment with 0.3 µM LatA (*n * =  3)

Gene Deletion Mutant	Uninucleate	Binucleate (Complete Septum)	Binucleate (Fragmented Septum)	Tetranucleate (Fragmented Septum)	Ratio (Fragmented/Nonfragmented)
*ain1*Δ	69 ± 14	3 ± 3	28 ± 15	0	0.39
*ppk4*Δ	44 ± 29	1 ± 1	52 ± 27	4 ± 6	1.16
*exo2*Δ	21 ± 12	2 ± 2	76 ± 11	1 ± 2	3.30
*erg5*Δ	53 ± 36	2 ± 3	43 ± 37	1 ± 1	0.78
SPAC3H8.08c	68 ± 18	5 ± 7	27 ± 11	0	0.36
*mvp17*Δ	19 ± 14	7 ± 6	70 ± 9	4 ± 3	2.66
*pabp*Δ	23 ± 5	0	72 ± 4	4 ± 1	3.10
*tup12*Δ	43 ± 31	3 ±5	54 ± 35	1 ± 1	1.18
*acp2*Δ	66 ± 20	1 ± 1	33 ± 20	0	0.50
*end4*Δ	60 ± 19	1 ± 1	34 ± 14	1 ± 2	0.56
*cch1*Δ	41 ± 31	2 ± 2	56 ± 31	0	1.30
*cat1*Δ	51± 18	3 ± 6	43 ± 14	2 ± 3	0.79
SPAC9.02c	46 ± 27	5 ± 6	47 ± 34	0	0.92
*pka1*Δ	62 ± 14	3 ± 4	32 ± 9	2 ± 3	0.48
*imp2*Δ	10 ± 5	0	69 ± 2	21 ± 5	7.14
*adn2*Δ	24 ± 20	1 ± 2	72 ± 19	3 ± 3	2.83
*mni1*Δ	65 ± 11	7 ± 3	28 ± 7	0	0.39
*git11*Δ	54 ± 19	1 ± 2	44 ± 21	0	0.81
*ngg1*Δ	22 ± 4	2 ± 3	75 ± 6	1 ± 1	3.14
*but2*Δ	63 ± 9	2 ± 3	34 ± 6	0	0.52
*kin1*Δ	18 ± 5	11 ± 9	63 ± 14	8 ± 2	2.16
*tra1*Δ	47 ± 12	8 ± 7	43 ± 12	2 ± 2	0.79
SPCC1020.07	47 ± 32	3 ± 1	49 ± 30	1 ± 1	0.99
*git3*Δ	53 ± 21	0 ± 0	45 ± 21	2 ± 2	0.84
SPCC4F11.03c	31 ± 14	6 ± 9	58 ± 21	5 ± 2	1.54
*snd1*Δ	39 ± 12	5 ± 6	55 ± 17	1 ± 1	1.26
SPCC70.06	41 ± 20	5 ± 5	52 ± 26	2 ± 2	1.12
*efr3*Δ	32 ± 17	2 ± 2	66 ± 18	0	1.91
*upf1*Δ	39 ± 27	3 ± 3	58 ± 29	1 ± 1	1.38
SPAC3C7.07c	54 ± 10	9 ± 2	35 ± 12	2 ± 3	0.56
*pex14*Δ	70 ± 8	8 ± 8	22 ± 5	0	0.28
*git5*Δ	55 ± 16	2 ± 2	42 ± 19	2 ± 3	0.74
*apm3*Δ	48 ± 5	3 ± 1	48 ± 3	1 ± 1	0.94
SPBC725.10	53 ± 24	1 ± 2	45 ± 23	0	0.82
*ppk33*Δ	58 ± 8	5 ± 5	37 ± 3	0	0.60
*cgs2*Δ	60 ± 4	4 ± 4	36 ± 6	0	0.56
SPCC297.05	59 ± 27	2± 3	38 ± 28	0	0.63
SPCC794.03	60 ± 16	7± 6	31± 14	1 ± 2	0.47
*pap1*Δ	46 ± 11	1 ± 1	53 ± 11	0	1.14
Wild type	88 ± 3	12 ± 3	2 ± 1	0	0.02

### GO enrichment analysis

Manual inspection of the results of the primary screen revealed a diverse set of genes with a variety of unique functions. To aid in extracting biologically meaningful information from this list, and to help form a foundation for developing future testable hypotheses, we next made use of the BiNGO tool. BiNGO is an open-source software package that can be used to uncover statistically significant enrichments in gene ontologies (Maere *et al.* 2005). A summary of the most compelling findings are discussed below and shown in Table 4. The full output of the analysis is included as File S2.

**Table 4:** Gene Ontology (GO) term enrichments identified by BiNGO using the primary screen gene list as input

Category Name	GO ID	Gene Set Name	Corrected p-Value	Genes
Cellular Component	GO:0000124	SAGA Complex	6.67 × 10^−5^	*gcn5*, *ada2*, *sgf29*, *tra1*, *spt8*, *spt20*, *ngg1*, *ubp8*
Cellular Component	GO:0016272	Prefoldin Complex	9.60 × 10^−4^	*gim4*, *bob1*, *gim3*, *gim1*, *pac10*
Cellular Component	GO:0034967	Set3 Complex	1.64 × 10^−3^	*hif2*, *snt1*, *set3*, *hos2*
Cellular Component	GO:0030123	AP-3 Adaptor Complex	1.64 × 10^−3^	*apm3*, *aps3*, *apl5*, *apl6*
Biological Process	GO:0016570	Negative Regulation of Transcription by Glucose	3.01 × 10^−6^	*pyp1*, *git3*, *git5*, *git11*, *gpa2*, *pka1*, *tup12*
Biological Process	GO:0045761	Regulation of Adenylate Cyclase Activity	1.64 × 10^−3^	*git3*, *git5*, *git11*, *cgs2*, *gpa2*, *cap1*
Biological Process	GO:006338	Chromatin Remodeling	4.17 × 10^−3^	*spt8*, *ubp8*, *hif2*, *sgf29*, *spt20*, *eaf1*, *ngg1*, *gcn5*, *ada2*, *tra1*, *hos2*, *snt1*, *pst2*, *jmj1*, *set3*, *rrp1*, *arp6*, *abo1*, *pcu4*, *snf5*, *tup12*
Biological Process	GO:007021	Tubulin Complex Assembly	2.22 × 10^−3^	*gim4*, *bob1*, *gim3*, *gim1*, *pac10*
Molecular Function	GO:0003712	Transcription Cofactor Activity	1.81 × 10^−3^	*spt8*, *ada2*, *spt20*, *snd1*, *sub1*, *pst2*, SPAC1A6.01c

#### Cellular component:

**Figure 5:**
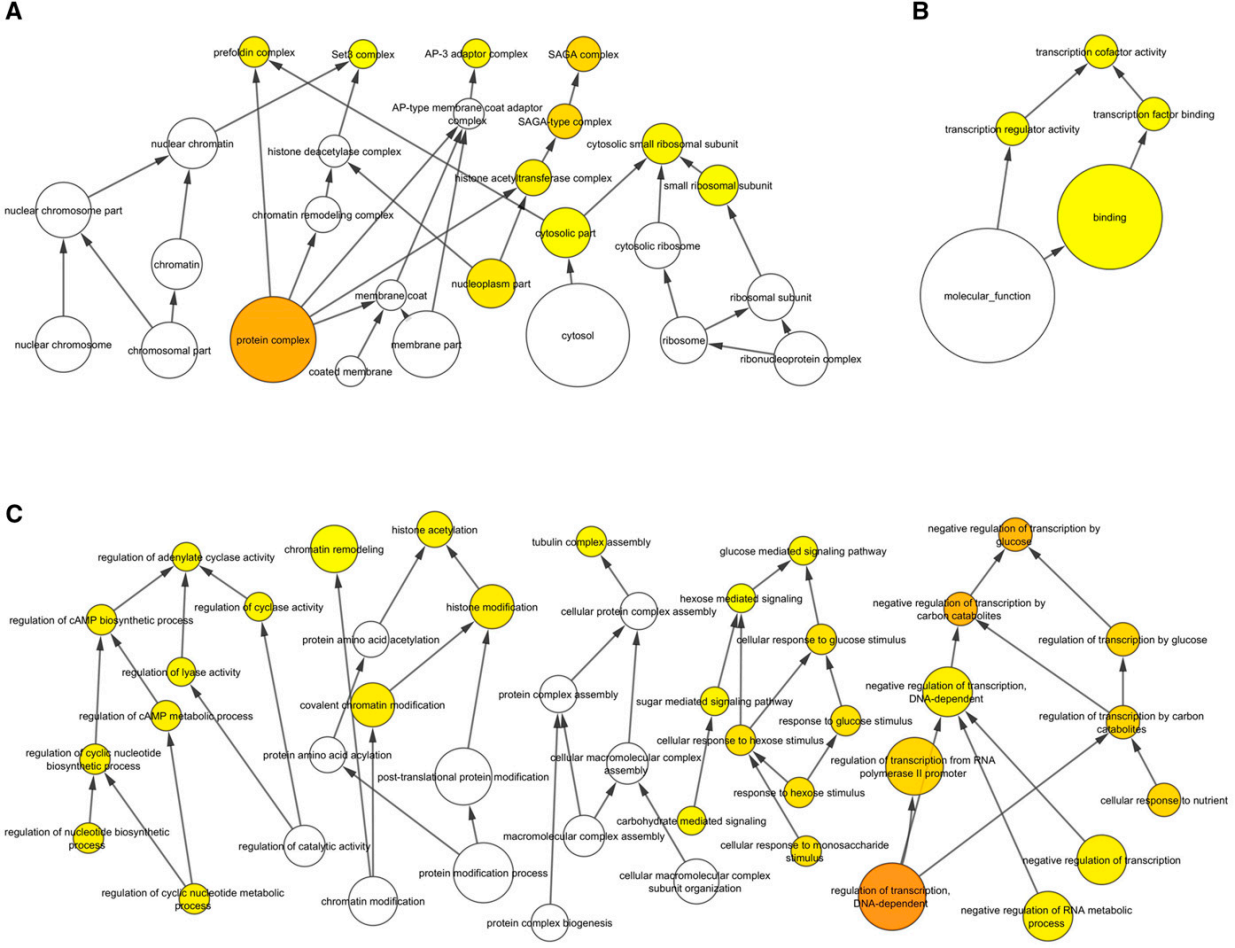
BiNGO GO term enrichment analysis visualized with Cytoscape using a hierarchical layout. The 288 hits of the primary screen were used as input against a background of the 3400 *S. pombe* genes of the Bioneer deletion set. Significant enrichments (p < 0.005) are highlighted in yellow. (A) GO Cellular Component. (B) GO Molecular Function. (C) GO Biological Process.

Under the category of Cellular Component, members of four protein complexes were found to be enriched in the submitted list relative to the background list: 1) the SAGA complex (eight hits out of 19 members), 2) the prefoldin complex (five hits out of six members), 3) the AP-3 adaptor complex (four hits out of four members), and the Set3 complex (four hits out of four members) (Figure 5A and Table 4). Interestingly, members of the Set3 complex have been previously characterized with respect to their role in responding to LatA-induced stress in *S. pombe* (Rentas *et al.* 2012) and will not be discussed further here. Of the remaining hits, only the prefoldin complex has a clear relationship to the actin cytoskeleton; being that it acts as a molecular chaperone and is required for the proper folding of actin monomers (Lundin *et al*. 2010). These facts suggest that the LatA sensitivity of prefoldin mutants is a reflection of reduced actin-directed chaperone activity, and furthermore, that this reduced activity is more acutely felt in the presence of LatA, which acts to reduce the pool of free actin monomers. It should also be noted that the gene products comprising the prefoldin complex were identified under the GO term “tubulin complex assembly” in the Biological Process category. This is because the prefoldins are also involved in regulating tubulin function. The critical prefoldin target(s) with respect to the cell’s response to LatA remains unknown.

The SAGA complex has a clearly defined role in histone modification and is also known to play a key role in regulating gene expression in response to stress (Rodríguez-Navarro 2009; de Nadal *et al.* 2011). For example, it is required for the upregulation of gene expression in response to heat shock, glucose limitation, as well as DNA damage (Huisinga and Pugh 2004; Geng and Laurent 2004; Ghosh and Pugh 2011). The present data suggest that components of the SAGA complex might also be required to properly modulate gene expression under LatA-induced cytoskeletal stress.

Lastly, the AP-3 adaptor complex plays an important role in the intracellular trafficking of membrane-associated proteins in eukaryotes (Dell’Angelica 2009). A role in regulating the actin cytoskeleton or cytokinesis has not been demonstrated in yeast. However, it is interesting to speculate, given that transport from the Golgi to the site of cell division is important for actomyosin ring stability in *S. pombe* (Arasada and Pollard 2014), whether the AP-3 complex might be involved in regulating cell division through modulating intracellular transport processes.

#### Molecular function:

Under the category of Molecular Function, BiNGO identified genes associated with the GO term “transcription cofactor activity” (Figure 5B and Table 4). Such cofactors function to transmit regulatory signals between gene-specific activators and the basal transcription machinery and are often associated with fine-tuning gene expression in response to environmental or developmental stimuli (Mannervik *et al.* 1999; Thomas and Chiang 2006). This finding is consistent with previous work showing large scale transcriptional changes upon LatA treatment (Saberianfar *et al.* 2011; Rentas *et al.* 2012), and suggests that these cofactors may play a role in coordinating or fine-tuning these shifts in transcriptional output.

#### Biological process:

Under the category of Biological Process, BiNGO identified genes associated with GO terms related to “chromatin remodeling” (21 genes), “negative regulation of transcription by glucose” (seven genes), and “regulation of adenylate cyclase activity” (six genes) (Figure 5C). Of the genes involved in chromatin remodeling, eight were components of the SAGA complex, and four were components of the Set3 complex. As stated above, it is well established that the Set3p complex plays a role in modulating the transcription of stress-responsive genes in response to LatA (Rentas *et al.* 2012). Whether the SAGA complex, through its histone modification activity, might also play a role in modulating transcription in response to LatA, remains to be determined.

Perhaps the most surprising enrichments were those involving glucose-mediated signaling. Of the genes enriched in the negative regulation of transcription by glucose and regulation of adenylate cyclase activity categories, the majority comprised members of the glucose/cAMP signaling pathway (*git3*, *git5*, *git11*, *gpa2*, *pka1*, *cgs2*). While this pathway is not known to regulate cytokinesis directly, it is required for invasive filament formation, which in turn involves the reorganization of proteins involved in regulating cell polarity and actin dynamics (Amoah-Buahin *et al.* 2005; Dodgson *et al.* 2010). Again, it is interesting to speculate as to whether the pathway might also be required to trigger the reorganization of actin regulators in response to LatA treatment.

With respect to the glucose/cAMP pathway, it is also interesting to note the conspicuous absence of the *git1* gene as a hit in the screen. Loss of *git1* results in an even stronger loss of adenylate cyclase activity than conferred by the loss of the *git3*, *git5*, or *git11* genes, and would therefore be expected to confer LatA sensitivity. Interestingly, upon closer inspection of the Bioneer *git1*Δ strain, we found that it did not display the semi-wee phenotype characteristic of the mutant. We obtained an independent version of the *git1* deletion mutant (Kao *et al.* 2006) and assayed its response to LatA. Interestingly, we found that this strain did indeed display a semi-wee phenotype, as well as a strong sensitivity to low doses of LatA (data not shown). We conclude that misregulation of the glucose/cAMP pathway does generally lead to cytokinesis failure in the presence of LatA.

While the formal testing of the hypotheses suggested above is beyond the scope of this report, the results of this bioinformatical analysis, together with the other results of this study, make it clear that a diverse set of regulatory modules, representing a wide-ranging array of biological functions, are required to properly respond to LatA-induced cytoskeletal perturbations. While genes involved in regulating cytokinesis and/or the actin cytoskeleton are indeed represented (*e.g.*, the α-actinin gene, *ain1*, the F-actin capping subunit, *acp2*, and the contractile ring protein, *imp2*) (Li *et al.* 2016; Nakano and Mabuchi 2006; Ren *et al.* 2015), other functions, ranging from histone modification to intracellular transport to glucose-mediated signaling are also implicated. How these modules interact and communicate to create a cellular state conducive to cytokinesis remains to be determined and will undoubtedly require further experimentation using a broad, multidisciplinary, and systems-based approach.

### Summary

Having previously shown that low dose LatA treatment can be used as an effective tool to perturb the cell division machinery (Mishra *et al.* 2004; Karagiannis 2012; Mishra *et al.* 2005; Karagiannis *et al.* 2005; Karagiannis and Balasubramanian 2007; Saberianfar *et al.* 2011; Rentas *et al.* 2012), we engaged in a genome-wide screen to identify gene deletion mutants that are hyper-sensitive to the drug. In this manner we hoped to identify novel genetic regulators that function to promote the faithful and reliable execution of cytokinesis in fission yeast. Out of 3400 strains, we identify and validate a set of 38 gene deletion mutants, representing a diverse set of biological functions, which are prone to cytokinesis failure in the presence of LatA. Importantly, while these mutants display cytokinesis defects in the presence of the drug, they are nonetheless fully capable of cell division when not stressed. These results suggest that fission yeast employ a diverse set of regulatory modules to actively counter cytoskeletal perturbations, thereby ensuring cell survival and proliferation.

## 

## Supplementary Material

Supplemental Material
