# Supplementary material for: A Genetic Screen for Fission Yeast Gene Deletion Mutants Exhibiting Hypersensitivity to Latrunculin A
Source: G3 (Bethesda). 2016 Jul 27;6(10):3399–408. doi: 10.1534/g3.116.032664 (PMC5068959; doi:10.1534/g3.116.032664)
Supplement: Supplemental Material [file supp_6_10_3399__index.html]

A Genetic Screen for Fission Yeast Gene Deletion Mutants Exhibiting Hypersensitivity to Latrunculin A — Supplemental Material 

# A Genetic Screen for Fission Yeast Gene Deletion Mutants Exhibiting Hypersensitivity to Latrunculin A

## Supplemental Material for Asadi, Michalski, and Karagiannis, 2016

**Files in this Data Supplement:**

- Figure S1 - Overview of the genetic screen described in this study. (.tif, 9 MB)
- File S1 - Primary screen hits categorized into high, medium, and low-confidence groups. (.xlsx, 99 KB)
- File S2 - BiNGO GO term enrichment analysis for the 288 hits of the primary screen. (.xlsx, 24 KB)
