# Supplementary figures and images for: A Genetic Screen for Fission Yeast Gene Deletion Mutants Exhibiting Hypersensitivity to Latrunculin A
Source: G3 (Bethesda). 2016 Jul 27;6(10):3399–408. doi: 10.1534/g3.116.032664 (PMC5068959; doi:10.1534/g3.116.032664)

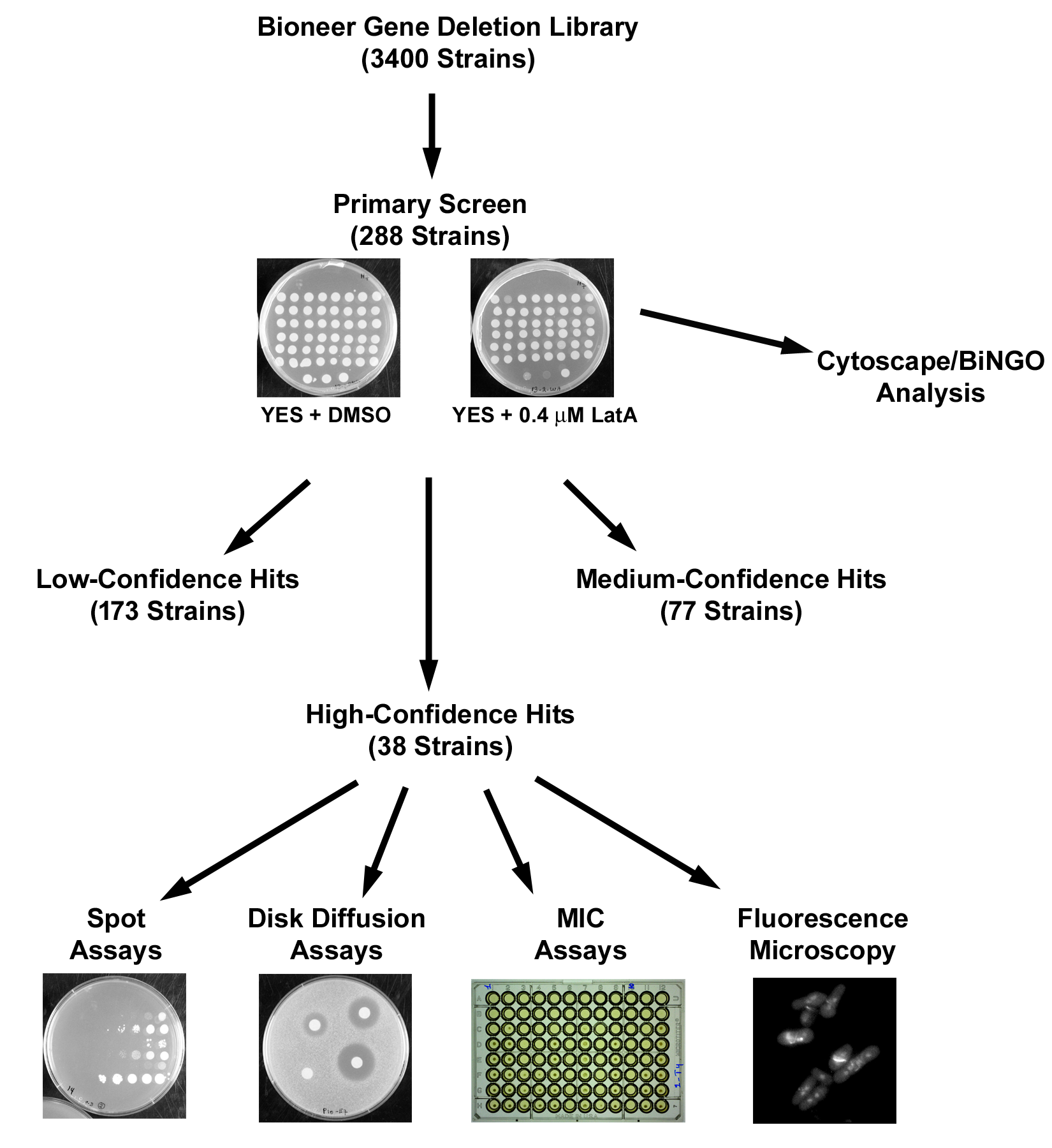

Supplement: Supplemental Material [file supp_g3.116.032664_FigureS1.tif]
